# Economic Distress of Breast Cancer Patients Seeking Treatment at a Tertiary Cancer Center in Mumbai during COVID-19 Pandemic: A Cohort Study

**DOI:** 10.31557/APJCP.2021.22.3.793

**Published:** 2021-03

**Authors:** Tabassum Wadasadawala, Soumendu Sen, Rakesh Watekar, Pallavi Rane, Rajiv Sarin, Sudeep Gupta, Vani Parmar, Sadhana Kannan, Sanjay K Mohanty

**Affiliations:** 1 *Tata Memorial Center, Homi Bhabha National Institute, Mumbai, India. *; 2 *International Institute for Population Sciences, Mumbai, India. *

**Keywords:** Economic burden, breast cancer, pandemic, distress financing, India

## Abstract

**Background:**

Cancer treatment during nationwide lockdown due to the COVID-19 pandemic has posed several challenges in the delivery of cancer care and carries tremendous potential sequel of impoverishing the households. This study aims to examine the economic distress faced by breast cancer patients receiving treatment at Tata Memorial Center (TMC) Mumbai, India during the nationwide lockdown initiated in March 2020 following the outbreak of COVID-19.

**Methods:**

A total of 138 non-metastatic breast cancer patients who were accrued in this study at TMC before imposing of lockdown, and their treatment was impacted because of the COVID-19 outbreak, were interviewed. Telephonic interviews were conducted using a structured schedule which contained information on household and demographic characteristics of the patients, knowledge about COVID-19, their daily expenditure for treatment, difficulties faced during lockdown and how they met expenditures. Descriptive statistics and logistic regression were used in the analyses.

**Results:**

The average monthly expenditure of cancer patients had increased by 32% during the COVID-19 period while the mean monthly household income was reduced by a quarter. More than two-thirds of the patients had no income during the lockdown. More than half of the patients met their expenditure by borrowing money, 30% of the patients used their savings, 28% got charity and 25% used household income. About 81% of the patients had reported shortage of money, 32% reported shortage of food and 28% reported shortage of medicine. The distress financing was significantly higher among patients receiving treatment in Mumbai compared to those receiving treatment at their native cities (67% vs. 46%), patients under 40 years of age, illiterate, currently married, Muslim and staying at a rented house.

**Conclusion:**

The incremental expenditure coupled with reduced or no income due to the closure of economic activities in the country imposed severe financial stress on breast cancer patients.

## Introduction

Cancer has become the leading cause of death among non-communicable diseases in at least half of the countries worldwide. Cancer accounted for 233.5 million Disability Adjusted Life Years (DALYs) in 2017 Among all cancer related DALYs 97% was attributed to Years of Life Lost (YLL) and 3% was attributed to Years of Live with Disability (YLD) (Fitzmaurice et al., 2019). Of the 18.1 million cancers diagnosed globally in 2018, about 2.1 million were of breast cancer. Breast cancer is the leading type of cancer in 80% of the countries and the leading cause of cancer mortality in the majority of the countries (Bray et al., 2018).The incidence and mortality from cancer is increasing globally and is compounded by the population growth, population ageing and changing lifestyle (Pramesh et al., 2014). Besides, deteriorating health and psychological stress, cancer patients and their caregivers undergo economic distress and often catastrophic health spending due to the high cost of treatment and lack of financial protection (Mishra et al., 2020). Access to affordable cancer care is the biggest challenge for developing economies like India (Pramesh et al., 2014). About 79% of Indian households face catastrophic health expenditure due to cancer and 46% of the expenses for cancer treatment is met through distress financing (Kastor and Mohanty, 2018). Mean out-of-pocket payment (OOP) on hospitalization is highest for cancer and the cost of cancer inpatient care in a private facilities is at least three times higher than public facility (Nair et al., 2013; Rajpal et al., 2018). Among all types of cancer, breast cancer accounts for 14% of total cases with an age-standardized mortality rate of 61.4%. Breast cancer is the most common cancer in India accounting for 14% of new cases in both gender and 27.7% cases among females . It ranks first in cancer mortality accounting for 11.1% of all cancer deaths in contrast to the 6.6% global estimates (Bray et al., 2018). 

Since December 2019, the world has witnessed a massive outbreak of COVID-19 that has become a global pandemic in a span of three months. The disease was triggered by the severe acute respiratory syndrome coronavirus 2 (SARS-CoV-2) originated in Wuhan province of China. The fatality rate is very high for the elderly, people with co-morbidities, lung cancer, males and patients undergoing immunosuppressive chemotherapy for cancer treatment (Li et al., 2020; Liang et al., 2020; Yang et al., 2020). The COVID-19 pandemic posed numerous challenges in cancer treatment as management of covid-19 gained priority over cancer care due to limited medical resources (Papautsky et al., 2020; Kutikov et al., 2020). The Asian National Cancer Centers Alliance (ANCCA) recently published the results of a survey conducted on the nuances of cancer care during COVID times within Asian countries and highlighted the role of various regional leadership strategies in safeguarding the health of staff and patients. (Gatellier et al., 2020). At the household level, the impact of COVID-19 is multilayered; loss of household income, temporary or permanent closure of business and loss of productivity (Baddour et al., 2020).

Patients with breast cancer receive multimodality treatment which comprises of breast surgery, systemic therapy in the form of chemotherapy, hormone therapy or targeted therapy and radiotherapy. This entails a prolonged duration of treatment which generally takes six months but can be delayed subject to the availability of facilities, financial background and waiting lists at public hospitals. 

The cancer treatment centers in India are very limited. Most of these centers are either located in metropolises or the capital city of the states thereby mandating patients to travel long distances for treatment. While cancer affects the rich and poor, the economic burden of cancer is very high for the poor and middle-class families. The recent COVID-19 pandemic has worsened the situation for patients suffering from cancer; the pandemic and the nationwide lockdown has now made them more vulnerable (Sharma and Pinato, 2020). Though studies have examined the impact of altered treatment protocols and severity of the disease among cancer patients, no attempt has been made to understand the economic hardship of cancer patients who are undergoing treatment during the COVID-19 pandemic. In this context, the main objective of this study is to examine the economic distress of breast cancer patients undergoing treatment at Tata Memorial Center, Mumbai during the COVID-19 pandemic and lockdown. 

## Materials and Methods

*Study population*

This study has been conducted by Tata Memorial Center (TMC) in collaboration with International Institute for Population Sciences (IIPS), Mumbai. A team of oncologists, health economists, statisticians, demographers and social workers designed and executed the study. This paper is based on 138 non-metastatic breast cancer patients who were accrued in this study at TMC before lockdown and continued their treatment during lockdown. A structured questionnaire was prepared and telephonic interviews were conducted. The questionnaire covered household, demographic and socio-economic characteristics including income and detailed record of the direct and indirect health expenditure of each visit to the hospital as well as inpatient care. This paper is derived from a project on economic impact of breast cancer patients that received approval from the institutional ethics committee and is registered on the Clinical Trial Registry of India (CTRI/2019/07/020142). All participants gave written informed consent for the study. 

*Outcome variables*

The primary outcome variables of interest are expenditure and income in pre COVID-19 and during the COVID-19 period and the extent of distress financing. Patients were asked about their daily expenditure on food, of cancer patients undergoing treatment at TMC accommodation, medicine, transport and others along with household income. Daily expenditure on food was defined as the expenditure incurred by patients and the accompanying persons on food during the treatment at lockdown period; accommodation cost was defined as the daily cost of renting rooms for the patients who came from their native places; medicine cost contained expenditure at the hospital for surgery, chemotherapy, radiotherapy, consumable medicines, investigation cost, registration and admission charges; cost incurred to reach hospital from current address has been captured under transport cost. Monthly expenditure includes the expenditure of the patients and consumption the accompanying person(s) at the place of treatment and it does not include expenditure of other household members at their permanent residence. Pre COVID-19 and COVID-19 period were demarcated by the day of starting of nationwide lockdown (before 24th March 2020 as pre COVID-19 and 24th March onwards as COVID-19) due to the virus outbreak. A patient was said to be incurring distress financing if she reported borrowing money from their friends or relatives to meet treatment and other expenditures. 

*Statistical analysis*

Descriptive statistics and logistic regression were used in the analyses. The distress financing variable has coded 1 if the patient met her expenditure through distress financing and 0 otherwise. The statistical analysis was done using STATA 13.

**Table 1:** Percent Distribution of Breast Cancer Patients Undergoing Treatment at TMC, March-June 2020

	Percentage	N*
Type of Patients		
General	64.49	89
Private	17.39	24
Non-chargeable	18.12	25
Place of Treatment		
Mumbai	71.74	99
Native city (other than Mumbai)	28.26	39
Age Distribution		
Below 40 years	31.88	44
More than 40 years	68.12	94
Median Age (years)	45	138
Permanent Residence		
Within state	31.16	43
Outside state	68.84	95
Financially Dependent (%)	97.1	138
Income during Pandemic (in Indian Rupees)		
No income	68.12	94
Less than 15,000	18.12	25
More than 15,000	13.77	19
Educational Attainment		
No education	24.64	34
Up to 5 years	15.94	22
6+ years	59.42	82
Mean Years of Schooling (years)	7.35	138
Marital Status		
Currently married	86.96	120
Others	13.04	18
Current Accommodation		
Own house/friends house	56.52	78
Rental	29.71	41
Ashram/ Gurudwar	13.77	19
Number of Accompanying Persons		
1	85.51	118
2 and more	14.49	20
Mean distance in kilometer from TMC	42.7	138
Total	100	138

*, Sample size of each category.

**Table 2:** Monthly Expenditure (in ₹) and Household Income (in ₹) of Breast Cancer Patients in Pre and COVID-19 Period, 2020

	Expenditure in Indian Rupees*			Income in Indian Rupees*		
	Pre COVID-19	COVID-19	% increase	Pre COVID-19	COVID-19	% decrease
Type of Patients						
General	13,173	23,744	80.2	17,942	4,022	77.6
Private	45,640	33,727	-26.1	96,206	26,680	72.3
Non-chargeable	9,475	16,464	73.8	2,490	708	71.6
Age of Patients						
Below 40 years	17,813	20,722	16.3	16,752	4,886	70.8
More than 40 years	18,692	25,955	38.8	35,557	8,798	75.2
Years of Schooling						
No schooling	12,585	19,383	54	11,240	2,147	80.9
Up to 5 years	11,772	13,650	16	9,648	2,500	74.1
6 years and above	22,609	29,174	29	42,203	11,146	73.6
Place of treatment						
Mumbai	12,648	20,901	65.3	29,137	3,232	88.9
Native City	33,043	32,882	-0.004	30,472	18,512	39.2
Permanent Residence						
Within Maharashtra	16,048	20,146	25.5	46,178	9,209	80.1
Outside of Maharashtra	19,481	26,161	34.3	22,151	6,800	69.3
Current Residence						
Own house	21,671	25,526	17.8	36,302	11,769	67.6
Rental house	16,499	23,368	41.6	28,405	2,854	90
Ashram/Gurudwar	9,158	21,182	131.3	4,420	368	91.7
Expenditure Tertile						
First (lowest)	7,680	16,560	115.6	13,564	2,479	81.7
Second	13,500	20,610	52.7	16,765	1,182	92.9
Third (highest)	34,290	35,850	4.5	58,086	18,935	67.4
Total	18,411	24,287	31.9	29,518	7,551	74.4
Median	12,000	13,500	NA	9,000	0	NA
Interquartile range	9,000	14,266	NA	17,660	5,000	NA

*=USD 1= ₹ 75.7111

**Figure 1:**
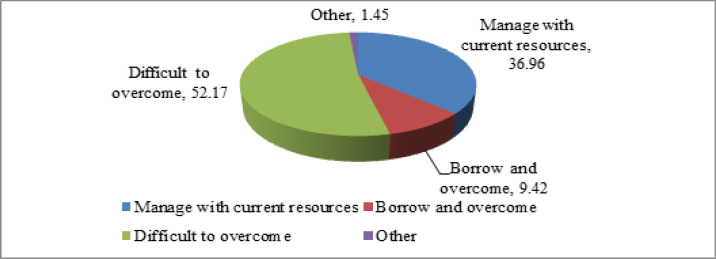
Strategy to Manage Financial Condition during COVID-19 Pandemic among Breast Cancer Patients, 2020

**Table 3:** Shortage of Money, Food, Medicine and Extent of Distress Financing (n and in %) during Lockdown Period, March- June 2020

	Shortage of money n (in %)	Shortage of food n (in %)	Shortage of medicine n (in %)	Shortage of any n (in %)	Distress financing n (in %)	N*
Type of Patients						
General	79 (88.7)	29 (32.58)	28 (31.46)	87 (97.75)	62 (69.66)	89
Private	10 (40)	0 (0)	4 (16)	24 (95.83)	6 (24)	25
Non-chargeable	23 (95.83)	15 (62.5)	7 (29.17)	24 (100)	16 (66.67)	24
Place of Treatment						
Mumbai	86 (86.87)	43 (43.43)	30 (30.3)	96 (96.97)	66 (66.67)	99
Native city	26 (66.67)	1 (2.56)	9 (23.08)	39 (100)	18(46.15)	39
Age of Patients						
Below 40 years	36 (81.82)	15 (34.09)	12 (27.27)	44 (100)	30 (68.18)	44
More than 40 years	76 (80.85)	29 (30.85)	27 (28.72)	91 (96.81)	54 (57.45)	94
Years of Schooling						
0 to 5 years	52 (92.86)	24 (42.86)	16 (28.57)	54 (96.43)	39 (69.64)	56
5 years and above	60 (73.17)	20 (24.39)	23 (28.05)	81 (98.78)	45 (54.88)	82
Marital Status						
Currently married	98 (81.67)	39 (32.5)	35 (29.17)	117 (97.5)	74 (61.67)	120
Others	14 (77.78)	5 (27.78)	4 (22.22)	18 (100)	10 (55.56)	18
Social Group						
SC/ST	21 (84)	8 (32)	5 (20)	25 (100)	14 (56)	25
OBC	49 (83.05)	22 (37.29)	14 (23.73)	57 (96.61)	37 (62.71)	59
Others	42 (77.78)	14 (25.93)	20 (37.04)	53 (98.15)	33 (61.11)	54
Permanent Residence						
Within Maharashtra	34 (79.07)	10 (23.26)	9 (20.93)	43 (100)	23 (53.49)	43
Outside of Maharashtra	78 (82.11)	34 (35.79)	30 (31.58)	92 (96.84)	61 (64.21)	95
Type of Current Residence						
Own house	59 (75.64)	10 (12.82)	17 (21.79)	78 (100)	42 (53.85)	78
Rental house	35 (85.37)	25 (60.98)	16 (39.02)	39 (95.12)	28 (68.29)	41
Ashram/Gurudwar	18 (94.74)	9 (47.37)	6 (31.58)	1 (5.74)	14 (73.68)	19
Expenditure Tertile						
First	44 (91.67)	19 (39.58)	9 (18.75)	46 (95.83)	32 (66.67)	48
Second	40 (90.91)	16 (36.36)	17 (38.64)	43 (97.73)	35 (79.55)	44
Third	28 (60.87)	9 (19.57)	13 (28.26)	46 (100)	17 (36.96)	46
Total	112 (81.16)	44 (31.88)	39 (28.26)	135 (97.83)	84 (60.87)	138

*, Sample size in each category

**Figure 2:**
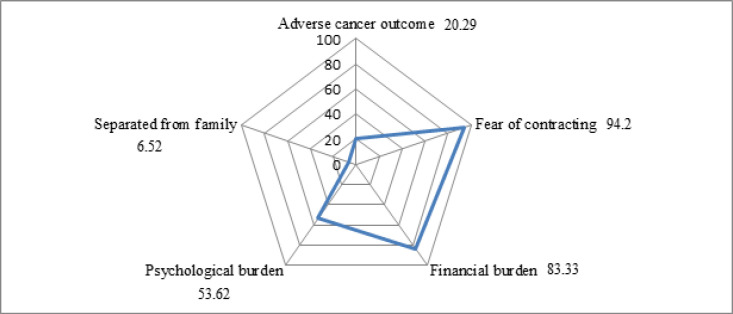
Main Concerns Regarding COVID-19 Pandemic among Breast Cancer Patients, 2020

**Table 4:** Coping Mechanism of Breast Cancer Patients at TMC, March-June 2020

Meeting of Expenditure	Percentage	N*
Meeting Food Expenditure		
Household Income	23.19	138
Saving	23.91	138
Borrowing from friends/relatives	42.03	138
Charity	10.87	138
Meeting Accommodation cost		
Household Income	8.64	138
Saving	19.75	138
Borrowing from friends/relatives	59.26	138
Charity	12.35	138
Meeting Medical expenditure		
Household Income	18.66	138
Saving	15.67	138
Borrowing from friends/relatives	37.31	138
Charity	28.36	138
Meeting Transport		
Household Income	23.58	138
Saving	21.95	138
Borrowing from friends/relatives	52.85	138
Charity	1.63	138
For any Expenditure		
Household Income	25.36	138
Saving	30.43	138
Borrowing from friends/relatives	60.87	138
Charity	28.99	138
Received financial aid	19.71	138

**Table 5:** Odds Ratio and 95% CI of Distress Financing with Socio-Economic Correlates, 2020

	Odd Ratio (95% CI)
Type of patients	
Private ®	1
General	4.51 (1.30-15.67)**
Non-chargeable	2.38 (0.40-13.91)
Age of patients	
Below 40 years ®	1
Above 40 years	0.43 (0.17-1.11)*
Years of schooling	
6 years and above ®	1
No schooling	1.37 (0.46-4.11)
upto 5 yeras	0.9 (0.28-3.04)
Marital status	
Currently married ®	1
Others	0.74 (0.22-2.49)
Permanent Residence	
Within Maharashtra ®	1
Outside of Maharashtra	2.46 (0.86-7.00)*
Current residence	
Own house ®	1
Rental house	0.95 (0.35-2.62)
Ashram/Gurudwar	0.76 (0.18-3.19)
Expenditure Tertile	
First ®	1
Second	2.01 (0.69-5.75)
Third	0.32 (0.10-1.05)*

CI, confidence interval; ®, reference category; **, *, statistically significant, ** p<0.05, * p<0.1

## Results

Table 1 presents the socio-demographic profile of 138 patients undergoing treatment for breast cancer at TMC, Mumbai. Among 138 patients, 65% were general, 17% were private and 18% were non-chargeable. The non-chargeable patients are treated at highly subsidized cost while general patients are treated at partially subsidized rate and the private patients are charged at market rate. The categorization of a patient depends on their financial background thoroughly scrutinized by social workers entitled at the hospital. About two-thirds of interviewed patients were aged 40 or more and one-third was under 40 years. Over two-thirds of the patients came from outside of the state of Maharashtra for treatment. About 56% of patients were staying in their own house or friend’s house for which they did not pay rent while 43% of patients were paying rent. On average, the cancer patients stay in a 42 kilometers radius from the cancer care center at Mumbai.

Table 2 presents the monthly expenditure of cancer patient and accompanying person(s) and income of the patients’ household in pre COVID-19 and COVID-19 period. The expenditure includes expenditure on food, accommodation, medicine, transport and others. The average monthly expenditure in pre COVID-19 period was ₹18411 compared to ₹24,287 during the lockdown, resulting in an increase of 32%. Except for the cost of accommodation, the daily expense of medicine, food and transport was increased (appendix A1). On the other hand, the mean monthly household income of the breast cancer patients was ₹ 29,517 before lockdown and it reduced to ₹7,551 following the lockdown. The median income during COVID-19 period was zero which indicates that half of the patients had income. The increase in expenditure for general and no-chargeable patients during COVID-19 period was 80% and 74% respectively. However, for the private patients, there was a reduction in expenditure as most of them were referred to local cancer treatment centers and as a result, their accommodation and transport cost had been reduced. The monthly expenditure had been doubled for the patients who stayed in Ashram or Gurudwara and for poor patients. The reduction of income was almost even across all types of patients. The monthly household income had been reduced more than the two-third proportion of earlier income for almost all socio-economic strata. 

Table 3 presents the extent of distress financing by the socio-economic characteristics of the patients. About 81% of patients had faced a shortage of money, 32% had reported a shortage of food and 28% of the patients had reported a shortage of medicine. Patients who were non-chargeable, undergoing treatment at TMC, staying at Ashram or Gurudwara and financially dependent were facing more shortage of money than their other counterparts. Similarly, shortage of food was largely reported by non-chargeable patients, patients undergoing treatment at TMC, with no schooling, Muslim patients and patients who stay at the rental house. On the other hand, no strong variation in the shortage of medicine was observed across socio-economic strata. About 61% of patients had met their expenditure through distress financing. The extent of distress financing was highest among general patients (70%), followed by non-chargeable (67%) and it was lowest for private patients (24%). The distress financing was highly prevalent among TMC patients (67% vs. 46% among local), patients with age below 40 years, patients with no schooling, currently married, Muslim and staying at rented house.

Table 4 presents the coping mechanism of meeting the daily expenditure of breast cancer patients. More than half of the patients met their expenditure by borrowing money from friends or relatives. About 30% of the patients met their expenditure from savings followed by charity (28%) and household income (25%). During the pandemic, only 20% of the patients had received financial aid from any sources.

Table 5 presents the result of logistic regression on the determinants of distress financing of breast cancer patients. The odds of incurring distress financing was significantly higher among general patients (OR: 4.51; 95% CI: 1.30-15.67) and patients who were from outside of Maharashtra. The likelihood of incurring distress financing was significantly lower among patients age above 40 years (OR: 0.43; 95% CI: 0.17-1.11) and patients from third consumption tertile (OR: 0.32, 95% CI: 0.10-1.05). 

Strategy to manage financial condition during the COVID-19 pandemic among the patients are shown in Figure 1. Among 138 patients, more than half of the patients (52%) reported that this pandemic is very difficult to overcome for them. Only 37% of the patients agreed that they can manage with the current resources and 9% stated that they would be able to overcome the situation by means of borrowing. Figure 2 represents the radar chart of major concerns about the pandemic among breast cancer patients of TMC. Fear of contracting the virus (94%) was a major concern. More than one-fourth of the patients had shown concern over adverse cancer outcomes and separated from family.

## Discussion

Since the third week of March 2020, India has witnessed a nationwide complete lockdown due to the outbreak of COVID-19. Breast cancer patients of TMC, Mumbai has faced a double burden of economic hardship during the pandemic; one is due to the high catastrophic expenditure associated with cancer treatment and the other is due to financial instability resulting from lockdown. The salient findings of the study are as follow: 

First, more than two-thirds of the patients at TMC had come from outside of the state and 68% of the patients had no income during the lockdown which triggered the vulnerability of the patients. Second, the monthly expenditure of the patients had increased by about one-third of the pre-pandemic expenditure. On the other hand, income had reduced by three-fourths. General patients had the highest increment of expenditure followed by non-chargeable patients. However, the expenditure of private patients had reduced. Third, the daily expenditure was met mainly by borrowing from friends or relatives. About 61% of the patients had borrowed money from friends and relatives. This was even larger for the patients with no schooling (76.4%) and patients belong to the second tertile (80%). Fourth, the likelihood of incurring distress financing was higher among general patients and patients who came from outside of the state. Fifth, more than half of the breast cancer patients reported that it was difficult to overcome the current situation of pandemic and almost all of them had fear of contracting the virus. 

Here we have some possible explanations for our findings. Most of the patients were from outside of the state, and this is one of the reasons for bearing a higher cost of daily expenditure on food, accommodation and transport. The daily wage and lack of job security in informal sectors resulted in a loss of income during the lockdown which was evident in 68% of the households with cancer patients in our study cohort. General patients had suffered more as they had to bear the cost of the treatment which was higher than their current ability to pay. They receive lesser subsidy than non-chargeable patients which resulted in higher expenses. Patients who cannot afford to pay rents while undergoing treatments at TMC, generally search for some religious places where they can stay by paying a minimum amount or sometimes the service is provided free. Despite this there was a sub-group which suffered more economic hardship and medicine accounted for the major share in their expenditure. The increment of the monthly expenditure was almost even across all subgroups but with some exceptions. For instance, the expenditure of private patients had decreased as most of them (19 patients out of 25) had been transferred to their native places for treatment. On the other hand, patients who stayed at religious places, their expenditure was lowest in pre COVID-19 period but during COVID-19 period, the expenditure had increased to just below the average monthly expenditure. This explains the doubling of expenditure and subsequently the economic hardship faced by those patients. The cost of transport accounted a significant increase in expenditure. As public transport was completely shut down, patients had to take cabs with higher rental prices than the pre COVID period. The increment in expenditure coupled with reduced or no income due to the closure of economic activities in the country had left needy patients with no options other than borrowing money from friends or relatives. The distress financing adds another burden to the patient’s family apart from higher out-of-pocket expenditure and zero income. 

India is one of the worst hit nations from the coronavirus and currently ranked second in total number of infected populations. Mumbai is among the worst hit metro cities accounts 4% of the total COVID-19 positive cases in the country. It has led to the modification of treatment protocols to balance the spread of COVID-19 among patients and health care workers and cancer cure. During the pandemic, accessing treatment facilities was dramatically affected mainly due to the travel restrictions and also due to the operational restrictions at treatment facilities. (Goyal et al., 2020)

The importance of digital infrastructure in health care delivery has been felt significantly (Yadav et al., 2020). Till March 2020, there was no law of providing digital consults to the patients but after the lockdown, the Indian Ministry of Health allowed to provide the full potential of technology in health care delivery (De Guzman and Malik, 2020). Telemedicine is one of the ways forward to the current shortage of health care delivery. For cancer patients, telemedicine can be one of the major preventive strategies to keep them safe and out of fear. Although this does not replace in-person visits, it is the most beneficial substitute amid the shutdown of public transport (Lee et al., 2020; Sirintrapun and Lopez, 2018). Patients who were receiving treatment in their native cities were those patients who were provided teleconsultation to help them continue the treatment so that undue delay in treatment and its adverse impact is averted. India’s expenditure on health care is one of the lowest. Recently, the Government of India has introduced an ambitious health policy that will cover annual health care cost up to ₹ 500,000 per family. This is the “world’s largest health care initiative” that is expected to cover 500 million poorest in the country. However, following such economic hardship faced by households with cancer patients, especially in the time of the pandemic, there needs to be more clarity from policies to finance such high-cost disease. The policies need to prioritize the optimal treatment of cancer patients as well as protect the households from financial catastrophe. 

The findings of the study are based on a small sample size of 138 patients and may be difficult to generalize. However, important insights on the economic hardship of breast cancer patients during lockdown have come out from this study.

COVID-19 outbreak around the world has impacted the health care delivery system badly. Cancer patients have suffered the most due to their higher cost of treatment and loss of household income resulting from complete lockdown. Policies should prioritize coverage of the treatment cost as well as safe guarding the livelihood. Providing cancer care through digital infrastructure could be one of the ways forward to the current situation.

## Author Contribution Statement

Authorship contributions TW and SKM conceptualized the study, research design, drafted and edited the manuscript. SS analyzed the data and drafted the manuscript. RW collected the data. PR and SK contributed to data analysis and statistical review. RS VP and SG critically reviewed the manuscript. 
